# Emerging Trends in “Smart Probiotics”: Functional Consideration for the Development of Novel Health and Industrial Applications

**DOI:** 10.3389/fmicb.2017.01889

**Published:** 2017-09-29

**Authors:** Racha El Hage, Emma Hernandez-Sanabria, Tom Van de Wiele

**Affiliations:** Center for Microbial Ecology and Technology, Faculty of Bioscience Engineering, Ghent University, Ghent, Belgium

**Keywords:** FMT, next generation probiotics, bacterial consortium, synthetic community, CDI

## Abstract

The link between gut microbiota and human health is well-recognized and described. This ultimate impact on the host has contributed to explain the mutual dependence between humans and their gut bacteria. Gut microbiota can be manipulated through passive or active strategies. The former includes diet, lifestyle, and environment, while the latter comprise antibiotics, pre- and probiotics. Historically, conventional probiotic strategies included a phylogenetically limited diversity of bacteria and some yeast strains. However, biotherapeutic strategies evolved in the last years with the advent of fecal microbiota transplant (FMT), successfully applied for treating CDI, IBD, and other diseases. Despite the positive outcomes, long-term effects resulting from the uncharacterized nature of FMT are not sufficiently studied. Thus, developing strategies to simulate the FMT, using characterized gut colonizers with identified phylogenetic diversity, may be a promising alternative. As the definition of probiotics states that the microorganism should have beneficial effects on the host, several bacterial species with proven efficacy have been considered next generation probiotics. Non-conventional candidate strains include *Akkermansia muciniphila, Faecalibacterium prausnitzii, Bacteroides fragilis*, and members of the Clostridia clusters IV, XIVa, and XVIII. However, viable intestinal delivery is one of the current challenges, due to their stringent survival conditions. In this review, we will cover current perspectives on the development and assessment of next generation probiotics and the approaches that industry and stakeholders must consider for a successful outcome.

## Introduction

The gut microbiota plays a significant role in human health, participating in several functions beneficial to the host (Patel and DuPont, 2015; Kristensen et al., 2016). It has been implicated in preventing pathogen colonization (Hand, 2016), shaping our immune system (Round and Mazmanian, 2009; Patel and DuPont, 2015; Macpherson et al., 2017), stimulating the production of gastrointestinal hormones (Saulnier et al., 2013), and regulating brain behavior (De Palma et al., 2014, 2017) through production of neuroactive substances (Steenbergen et al., 2015; Kristensen et al., 2016).

Additionally, the gut microbiota has been involved in the fermentation of non-digestible carbohydrates reaching the colon. This process leads to the production of short chain fatty acids (SCFAs), which elicit health benefits (den Besten et al., 2013). The human gut microbiota can be manipulated through either passive or active processes. Passive factors include hygiene, lifestyle, and diet. For instance, primary colonizers of the gut involved in the immune development are shifted by sanitary practices (Zhou, 2016). In addition, dietary constituents can promote phylogenetic variations in the microbiota (Graf et al., 2015). In this context, prebiotics are defined as “a substrate that is selectively utilized by host microorganisms conferring a health benefit” (Gibson et al., 2017). Prebiotics act as growth substrates (Patrascu et al., 2017) to enhance the activity of bacterial genera (Scott et al., 2015) such as bifidobacteria and butyrate-producing clostridia (Rivière et al., 2016). SCFA and vitamins resulting from the fermentation of these components are crucial for human health (Graf et al., 2015). In terms of lifestyle factors, physical activity is known to positively impact the diversity of gut microbiota. In fact, gut microbiota of athletes is more diverse than that of non-athletic subjects (Clarke et al., 2014). Amongst the active processes manipulating microbiota composition are antibiotics and probiotics. Antibiotic use has been linked to dysbiosis (Langdon et al., 2016), even leading to low diversity, evenness, and taxonomic richness (Dethlefsen and Relman, 2010; Francino, 2016). Moreover, presence and expression of microbial genes are altered following antibiotic therapy (Reijnders et al., 2016). These detrimental outcomes may lead to decreased SCFA, glycolysis, vitamin production, homeostasis of the immune system, and impaired protection against pathogens (Guarner and Malagelada, 2003). As a result, antibiotic associated diarrhea (AAD) and recurrent infectious diseases like *Clostridium difficile* infection (CDI) may occur (Francino, 2016).

On the other side of the spectrum are probiotics, which can affect the host either directly or through their products, or even influence the activity of resident bacteria in the host (Scott et al., 2015). Probiotics are defined as “live microorganisms which when administered in adequate amounts confer a health benefit on the host” (WHO/FAO, 2006; Hill et al., 2014). The effect of probiotics in preventing metabolic syndromes such as obesity, type 2 diabetes (Kasińska and Drzewoski, 2015), and dyslipidemia has been reported (Asemi et al., 2013). For instance, administration of *Bifidobacterium* (Yin, 2010; Chen et al., 2011; Plaza-Diaz et al., 2014; Reichold et al., 2014; Savcheniuk et al., 2014; Wang et al., 2014) and *Lactobacillus* species reduced body weight gain and adipose tissue in mice fed high-fat diet through stimulation of adiponectin production (Kim et al., 2013; Kobyliak et al., 2016). In addition, lactobacilli have been proven to have therapeutic effects in different pathologies (Di Cerbo et al., 2016). Moreover, probiotics regulate the mucosal immune response (Klaenhammer et al., 2012), improving the activity of macrophages (Sang, 2010) and changing the expression of the genes associated. Even though these outcomes depend on specific bacteria and strains, probiotics may interact with TLR and downregulate the expression of NF-κB and pro-inflammatory cytokines (Ng et al., 2009; Plaza-Diaz, 2014). For instance, peptides of microbial anti-inflammatory molecules (MAMs) that are found in the *Faecalibacterium prausnitzii* supernatant inhibit the NF-κB pathway *in vitro* and *in vivo* (Breyner et al., 2017), confirming the anti-inflammatory and therapeutic properties of *F. prausnitzii* (Martín et al., 2014). These properties and protective effects of *F. prausnitzii* were identified in different models such as dinitrobenzene sulfate (DNBS)-induced colitis model, dextran sodium sulfate (DSS)-induced colitis (Breyner et al., 2017), and 2,4,6-trinitrobenzenesulfonic acid (TNBS)-induced acute colitis in mice (Miquel et al., 2015). Additionally, levels of anti-inflammatory cytokines and immunoglobulins, immune cell proliferation, and production of proinflammatory cytokines produced by the T cells may be modulated following probiotic supplementation (Miettinen et al., 1996; Nazemian et al., 2016). Furthermore, probiotics can be alternative strategies for inflammatory disorders, as they upregulate the production of CD4+Foxp3+ regulatory T cells (T_regs_) (Kwon et al., 2010; Yan and Polk, 2011).

Different effects on the immune function may be species- and strain-related (Klaenhammer et al., 2012). It has been reported that probiotics have therapeutic effect on the central nervous system by reducing the intestinal inflammation. In this way, the regulation of HPA axis and the activity of the neurotransmitters may be improved (Wallace and Milev, 2017). Probiotics from *Bifidobacterium* and *Lactobacillus* genera are usually delivered through fermented products such as yogurts, milk, and cheeses, or they can be delivered as food supplements (Besseling-van der Vaart et al., 2016).

## Monostrain and Multistrain Probiotics

Probiotics have been categorized into monostrain or multistrain/multispecies products (Timmerman et al., 2004). Different studies have confirmed positive effects on health when multistrain probiotics are used, due to the symbiosis among strains (Timmerman et al., 2004). Strains in multispecies probiotics can be from different genera. For instance, the efficacy of the multispecies probiotic consortium VSL#3 (*Streptococcus thermophilus, Eubacterium faecium, Bifidobacterium breve, Bifidobacterium infantis, Bifidobacterium longum, Lactobacillus acidophilus, Lactobacillus plantarum, Lactobacillus casei*, and *Lactobacillus delbrueckii* subspecies *bulgaricus*) was proven for the treatment of ulcerative colitis (Venturi et al., 1999; Timmerman et al., 2004). Besides, VSL#3 supplementation in women with gestational diabetes mellitus (GDM) may help regulate inflammatory markers and positively influence glycemic control (Jafarnejad et al., 2016). In addition, Chapman et al. (2011) described that probiotic mixtures were more effective than single-strain probiotics in inhibiting pathogen growth and atopic dermatitis, suggesting further application on other diseases like IBD. Another multispecies probiotic called Ecologic^®^Tolerance/Syngut^TM^ was developed using four different probiotic strains (*Bifidobacterium lactis* W51, *L. acidophilus* W22, *L. plantarum* W21, and *Lactococcus lactis* W19). Strains of this consortium have been proven to strengthen the gut barrier function, have beneficial effects on post-immunological induced stress, inhibit Th2, and stimulate IL-10 levels, thus providing beneficial effects in patients with food intolerance (Besseling-van der Vaart et al., 2016). Moreover, a multispecies probiotic consortium, Ecologic AAD (*B. bifidum* W23, *B. lactis* W18, *B. longum* W51, *E. faecium* W54, *L. acidophilus* W37 and W55, *L. paracasei* W72, *L. plantarum* W62, *L. rhamnosus* W71, and *L. salivarius* W24), reduced diarrhea-like bowel movements when administered in healthy volunteers taking amoxicillin (Koning et al., 2008). Multispecies probiotics also prevented rise in fasting plasma glucose (FPG), to decrease high sensitivity C-reactive protein (hs-CRP), and to increase plasma glutathione (GSH) in diabetic patients (Asemi et al., 2013). van Minnen et al. (2007) provided evidence that manipulation of the intestinal flora with multispecies probiotics reduced bacterial translocation, morbidity and mortality in a rat model of acute pancreatitis. Furthermore, multispecies probiotics rapidly relieved IBS symptoms and shifted the microbiota composition (Yoon et al., 2013). According to these results, combining specific probiotic effects from diverse strains can lead to an additive and more synergetic multispecies probiotic consortium (Timmerman et al., 2007).

However, the phylogenetic origin of probiotics is currently limited to conventional formulations of *Bifidobacterium, Lactobacillus* species and other lactic acid bacteria (LAB) (Govender et al., 2013) or yeast strains. This may decrease the probiotic effectiveness in the prevention or therapy of diseases entailing severe dysbiosis. Hence, a functionally and phylogenetically diverse probiotic product may be desirable when alterations in the gut microbiota composition are present (Marotz and Zarrinpar, 2016). For instance, CDI and recurrent CDI are major medical conditions that need urgent treatment when conventional antibiotics fail. As a result, development of complex communities with targeted functions is needed.

## The Dilemma of Fecal Microbiota Transplant (FMT)

Fecal microbiota transplant (FMT) or fecal bacteriotherapy is an alternative strategy successfully used for the treatment of CDI (Kelly, 2013). Severe antibiotic therapy and CDI trigger dysbiosis, reducing diversity and functionality of the gut endogenous microbiota (Brandt, 2012). In this case, *C. difficile* spores can germinate, colonize, and thrive in the gut. Treatment of CDI requires additional antibiotics, increasing the risk of recurrent CDI (rCDI) after cessation of treatment, as a result of the dysbiosis caused by antibiotic therapy (Becattini et al., 2016; Francino, 2016), due to infection with the original strain (Barbut et al., 2000; Marsh et al., 2012) or re-infection caused by a different strain (Johnson et al., 1989; Kelly, 2009; Figueroa et al., 2012).

Poor colonization resistance from the gut microbiota and the patient’s poor immune response further contribute to CDI risk (Pérez-Cobas et al., 2015). Recurrent CDI risk is 10–20% after initial CDI (Surawicz et al., 2013), and it increases to 45% after a first relapse, and to 60% for those with two or more recurrences (Bartlett, 1990). However, FMT can resolve both CDI and rCDI (Bakken, 2009), with a success rate of 90% when further antibiotic treatments fail (Youngster et al., 2014; Rao and Safdar, 2015). Given the success of FMT, it is now being considered as potential treatment for disorders such as ulcerative colitis (Shi et al., 2016), irritable bowel syndrome (Distrutti et al., 2016), and metabolic syndrome (Hartstra et al., 2015). For instance, FMT induced remission in patients with active ulcerative colitis (Moayyedi et al., 2015), potentially as a result of the introduction of normal flora and the subsequent correction of the imbalance in the microbiota caused by the disease (Bakken et al., 2011). The complexity of the fecal sample can be the key factor behind the positive shift in the microbiota composition generated by the FMT (Marotz and Zarrinpar, 2016). Thus, diversity of the donor microbiome may be crucial (Leszczyszyn et al., 2016). Indeed, some patients do not respond to FMT, probably because only specific bacterial phylotypes can be therapeutic when effectively transferred (Vermeire et al., 2015). Hence, FMT efficacy for treating gastrointestinal disorders is controversial (Sbahi and Di Palma, 2016). Adverse effects after FMT include nausea, vomit, fever, abdominal pain, and diarrhea (Vermeire et al., 2015; Pigneur and Sokol, 2016). Data for long-term effects of FMT is lacking, but theoretically, any disease phenotype from the donor can be transferred to the patient (Sbahi and Di Palma, 2016). This could be expected, as the uncharacterized nature of FMT may result in undetected or unmonitored risk factors such as viruses, pathogens or even allergens being passed to the FMT recipient, causing disease. To overcome this problem, Petrof et al. (2013) developed a synthetic bacteria cocktail with characterized nature to substitute FMT. Alternatively, a thorough pre-screening should be performed on the donor before the actual procedure of the FMT. Thus, the French Group of Fecal microbiota Transplantation (FGFT) was created to secure and evaluate the practice in this field (Sokol et al., 2016). Despite having experience treating CDI, FMT is not yet the top treatment choice of physicians (Zipursky et al., 2014). However, the majority of gastroenterologists and physicians in metropolitan areas were supportive to the idea of creating a fecal transplantation center, and a high percentage of the physicians would refer their patients to those centers (Jiang et al., 2013).

## Alternatives for Fecal Microbiota Transplant (FMT)

Additional microbiome therapeutics using characterized microbial communities of selected fecal bacteria could be developed to replace FMT, and yield the desired outcome (Sbahi and Di Palma, 2016). For instance, Petrof et al. (2013) described a stool substitute constituted by 33 different purified intestinal bacteria isolated from a healthy donor (**Table 1**), to treat rCDI. In this study, the synthetic bacterial mixture was infused through the colon of the infected patient causing a change in the stool microbial profile. Major shifts reflecting the isolates of the synthetic mixture were still detectable 6 months after treatment. Thus, the concept of “RePOOPulate” the gut microbiome was coined. Authors of the study suggested that using a synthetic stool substitute may be an effective method to replace the use of FMT for treating rCDI. Although further validation is needed, complete resolution of the infection was achieved. Several advantages of this synthetic stool substitute can be highlighted. The composition of the administered bacterial cocktail is accurately characterized, facilitating registration. Further, assembly of the synthetic bacterial cocktail is highly reproducible enabling standardization and upscaling. In addition, patient safety can be guaranteed, because the bacterial mixture can be rendered pathogen- and virus-free (Petrof et al., 2013). These data suggest that a multi-species community such as that in the RePOOPulate study, can be more effective than single-strain probiotics or mixed cultures of probiotic species. This can be because the RePOOPulate community preserved its structure and thus successfully colonized a new environment (Petrof et al., 2013). Moreover, RePOOPulate consisted of a more phylogenetically diverse community including strains with beneficial health effects that can be candidates for next generation probiotics.

**Table 1:** Strains composing the RePOOPulate consortium.

Composition of Stool Substitute (RePOOPulate)
*Acidaminococcus intestinalis*
*Bacteroides ovatus*
*Bifidobacterium adolescentis (two different strains)*
*Bifidobacterium longum (two different strains)*
*Blautia producta*
*Clostridium cocleatum*
*Collinsella aerofaciens*
*Dorea longicatena (two different strains)*
*Escherichia coli*
*Eubacterium desmolans*
*Eubacterium eligens*
*Eubacterium limosum*
*Eubacterium rectale (four different strains)*
*Eubacterium ventriosum*
*Faecalibacterium prausnitzii*
*Lachnospira pectinoshiza*
*Lactobacillus casei/paracasei*
*Lactobacillus casei*
*Parabacteroides distasonis*
*Raoultella* sp.
*Roseburia faecalis*
*Roseburia intestinalis*
*Ruminococcus torques (two different strains)*
*Ruminococcus obeum (two different strains)*
*Streptococcus mitis*

## Next Generation Probiotics

Looking at its internationally recognized definition, probiotics are live microorganisms that, when administered in adequate numbers, confer health benefits on the host. Probiotics are usually isolated from our commensal gut bacteria, but cannot be given the definition of probiotics until their stability, content, and health effect are characterized (Sanders, 2008). Probiotics are thought to improve the balance in the host, prevent disturbances, and decrease the risk of pathogen colonization (Goldenberg et al., 2013). They have been referred to as functional foods or beneficial bacteria, and they have been considered for the prevention and treatment of *C. difficile*-associated diarrhea (CDAD) (Goldenberg et al., 2013). Probiotics can be found as capsules or food supplements in health food stores and supermarkets (Goldenberg et al., 2013). Pattani et al. (2013) reported that *Lactobacillus*-based formulations combined with antibiotics reduced the risk of AAD and CDI. They however, suggested that larger studies are needed to decide on the use of probiotic/antibiotic combination as a therapy over the single species probiotic (Pattani et al., 2013). Furthermore, findings from randomized control trials (RCTs) and meta analyses suggest that there is moderate evidence on the ability of probiotics to prevent primary CDI (people at risk of CDI), but there is no enough evidence suggesting the probiotics can prevent secondary CDI (recurrent CDI) (Evans and Johnson, 2015). There are still some evidence gaps for the use of probiotics in the prevention of CDI such as the interaction between specific classes of antibiotics with the probiotics used on CDI risk, the bacterial taxa that provides the best efficacy in the prevention of CDI, and the use of probiotics in immunocompromised or critically ill patients (Rao and Young, 2017). Hence, future RCT should consider these different concerns (Rao and Young, 2017). Besides, probiotics impact the gut-brain axis.

For example, *Bifidobacterium longum* NC3001 had beneficial effects on psychiatric comorbidities, which in turn could temporarily improve the quality of life in IBS patients, indicating that this probiotic reduces limbic reactivity (Pinto-Sanchez et al., 2017).

Overall, classical probiotics show limited effects on the human gut microbiota seeking the need for a better selection and formulation of bacterial strains (Neef and Sanz, 2013). Results from previous studies show promising outcomes in the treatment or prevention of diverse metabolic and inflammatory diseases by specific bacteria (Neef and Sanz, 2013). Those probiotics encompass species different from *Lactobacillus* and *Bifidobacterium* (Cani and Van Hul, 2015; Patel and DuPont, 2015). Nevertheless, the gut microbiome is a complex community, which makes it difficult to define the host–microbe interaction.

The United Nations Food and Agriculture organization (FAO) definition of probiotics is broad, allowing flexibility in terms of the phylogenetic origin of probiotics. Information generated from previous studies assisted in the selection of next generation probiotics, which include members from *Clostridium* clusters IV, XIVa and XVIII, *F. prausnitzii, Akkermansia muciniphila, Bacteroides uniformis* (Neef and Sanz, 2013; Patel and DuPont, 2015), *Bacteroides fragilis* (Round et al., 2011), and *Eubacterium hallii* (Udayappan et al., 2016). These next generation probiotics were evaluated in preclinical trials and yielded positive outcomes for inflammatory and metabolic disorders (Neef and Sanz, 2013; Patel and DuPont, 2015). In addition, new techniques are required for the development of new probiotic products containing strains from human origin. This is to say, these strains must come from the major groups of the intestinal microbiota, they have to be defined to have a safe status and proven to have potential beneficial effects (Martín et al., 2017). In the following sections, we will discuss some of the most promising bacterial species that are currently under consideration for being used as next-generation probiotics.

### Faecalibacterium prausnitzii

*Faecalibacterium prausnitzii* is an extreme oxygen sensitive (EOS) bacterium (Martín et al., 2017) belonging to the *Clostridium* cluster IV, and it accounts for 3–5% of the total fecal bacteria, and it is one of the predominant groups in the human feces (Breyner et al., 2017). Quévrain et al. (2016) reported low proportions of this species in the fecal and mucosa-associated microbiome in Crohn’s disease (CD). *F. prausnitzii* may possess *in vivo* and *in vitro* anti-inflammatory effects. *F. prausnitzii* may possess *in vivo* and *in vitro* anti-inflammatory effects. Breyner et al. (2017) confirmed the anti-inflammatory properties of MAM, and their ability to reduce Th1 and Th17 pro-inflammatory cytokines in Mesenteric Lymphatic Node (MLN) and colon tissues in both DNBS and DSS colitis model. MAM was also able to improve TGFβ cytokine which affects NF-κB activation in DNBS model thus protecting the host and decreasing intestinal inflammation (Breyner et al., 2017). In addition, *F. prausnitzii* can induce the *Clostridium*-specific IL-10-secreting regulatory T cell subset, present in several human colonic cells. Its capacity for lowering IL-12 and IFNγ production indicates that the interaction between *F. prausnitzii* and the host shape and maintain the gut barrier immune function (Quévrain et al., 2016). In this way, anti-inflammatory molecules from *F. prausnitzii* may be used as targeted anti-inflammatory drugs for CD. Moreover, MAM could function as a CD biomarker, predicting loss of *F. prausnitzii* functionality. However, further research should be conducted to elucidate the MAM production mechanisms, before considering it for CD management. Sokol colleagues reported that low proportions of *F. prausnitzii* on a resected ileal Crohn’s mucosa were associated with CD recurrence after 6 months. In addition, the oral administration of live *F. prausnitzii* or its supernatant in mice could reduce the severity of trinitrobenzene sulfonic acid (TNBS) colitis and correct the associated dysbiosis (Sokol et al., 2008). The results from this study suggest that *F. prausnitzii* can be considered as a promising probiotic candidate for the treatment of pathologies characterized by chronic gut inflammation (Sokol et al., 2008). Besides, all *F. prausnitzii* strains have proven anti-inflammatory properties, which allows them to further be tested in murine models to determine their beneficial effects before moving to human trials (Martín et al., 2017).

### Akkermansia muciniphila

Recent evidence shows that there is a link between the altered gut microbiota and metabolic diseases like obesity, diabetes mellitus, and cardiovascular disease (Schneeberger et al., 2015; Dao et al., 2016; Li et al., 2016). Higher abundance of *A. muciniphila*, a mucin degrading microbe, was associated with healthier metabolic status. Everard et al. (2014) and Schneeberger et al. (2015) studied the effects of high fat diet on metabolic parameters and the gut microbiota composition over time, and they found that *A. muciniphila* was decreased. The negative impact on *A. muciniphila* was associated with expression of lipid metabolism, inflammatory markers in adipose tissue, and different parameters like increased blood glucose, insulin resistance and plasma triglycerides (Schneeberger et al., 2015). This prompted the research toward investigating the putatively positive role of *A. muciniphila* in adipose tissue homeostasis and metabolism. Dao et al. (2016) assessed clinical parameters and *A. muciniphila* abundance before and after a 6-week calorie restriction period, followed by stabilization diet. The results of this intervention study indicate that the higher abundance of *A. muciniphila* at baseline was associated with improvement in blood glucose homeostasis, lipid profile, and body fat distribution after the intervention. Thus, *A. muciniphila* can be used as a prognostic tool for the success of diet interventions (Dao et al., 2016). Moreover, Li et al. (2016) reported that administration of *A. muciniphila* could reverse the atherosclerotic lesions, improve metabolic endotoxemia-induced inflammation, and ultimately restore the gut barrier.

### *Bacteroides fragilis* and *Bacteroides uniformis*

*Bacteroides* species are commensal bacteria that represent 25% of our gut bacterial population. They are gram negative, anaerobic, bile resistant, and non-spore forming bacteria. *Bacteroides* can be passed from the mother to the child during vaginal delivery, thus becoming primary colonizers of the gut. When retained in the gut, *Bacteroides* act as commensals and can be beneficial for the host (Wexler, 2007). The most common isolate from the clinical specimens is *B. fragilis*, which is the most virulent *Bacteroides* species (Wexler, 2007).

Bacterial colonization of the gut can greatly affect the immune system, either through the direct host–bacteria interaction, or by molecules produced by our commensal bacteria. *B. fragilis* produces polysaccharide A (PSA), which is an immunomodulatory molecule that activates the T-cell dependent immune responses (Troy and Kasper, 2010). Those responses are involved in the development and homeostasis of the host immune system (Troy and Kasper, 2010). Furthermore, Round et al. (2011) demonstrated that *B. fragilis* activates Toll-like receptor (TLR) pathways. This occurs because PSA signals through TLR2 on Foxp3+ (forkhead box P3) regulatory T cells to boost immunologic tolerance. As a result, PSA can be considered as a model symbiosis factor, because it preserves the balance between T cell types and maintains the immune system homeostasis (Round et al., 2011).

As for *Bacteroides uniformis* (*B. uniformis*) CECT 7771, it is considered a potential probiotic strain originally isolated from the feces of healthy breastfed infants. Oral administration of this specific strain in high fat diet-fed mice improved lipid profile, reduced glucose insulin and leptin levels, increased TNF-α production by dendritic cells (DCs) in response to LPS stimulation, and increased phagocytosis (Gauffin Cano et al., 2012). Thus, administration of *B. uniformis* CECT 7771 can ameliorate metabolic disorder and immunological dysfunction related to intestinal dysbiosis in obese mice (Gauffin Cano et al., 2012; Yang et al., 2016). Furthermore, acute administration of this strain to mice did not promote adverse effects on health status or food intake, and there was no bacteria translocation to blood, liver, or lymph nodes. This indicates that there are no safety concerns for this strain in mice, but further investigation should be completed in humans (Fernández-Murga and Sanz, 2016).

### Eubacterium hallii

*Eubacterium hallii* is an important anaerobic butyrate-producer resident in our gut, which influences the intestinal metabolic balance (Engels et al., 2016). Butyrate has been proposed to lower mucosal inflammation and oxidative status, strengthen the epithelial barrier function, and modulate intestinal motility in addition to being an energy source for colonocytes (Cani et al., 2011). *E. hallii* can yield propionate from a broad range of substrates. This versatility may enhance the host–gut microbiota homeostasis (Engels et al., 2016). Moreover, administration of *E. hallii* in obese and diabetic db/db mice increased energy metabolism and improved insulin sensitivity. However, increasing dosage of *E. hallii* did not impact body weight or food intake, indicating that this strain can a safe and effective alternative for insulin sensitivity (Udayappan et al., 2016).

## Cocktails of *Clostridium* Cluster IV and XIVa Members

As previously described, T_regs_ can regulate immune homeostasis and serve as a therapeutic target for different gut inflammatory disorders. Induction of the colonic T_regs_ is dependent on special properties of our commensal bacteria. *Clostridium* spp. belonging to clusters IV and XIVa (also known as *Clostridium leptum* and *coccoides* groups, respectively) are exceptional inducers of T_regs_ in the colon and can be considered as therapeutic options for IBD and allergies (Atarashi et al., 2011). Previous work indicated that a cocktail of strains isolated for the human gut microbiota can be more effective than a single strain in preventing or treating disease. Thus, Atarashi et al. (2013) isolated 17 strains belonging to Clostridia clusters XIVa, IV, and XVIII from a human fecal sample, which were effective in T_reg_ cell differentiation and accumulation in mouse colon. Authors proposed that the SCFAs produced by this community influenced the expression of *Foxp3*, a key gene controlling T_reg_ cell development (Atarashi et al., 2013). Incidentally, Clostridia clusters XIVa and IV are decreased in fecal samples from patients with inflammatory bowel disease (IBD), and thus the cocktail of the 17 strains could potentially reverse this dysbiosis (Atarashi et al., 2013).

## Industrial Applications and Interests

### Current Developments

Since the manipulation of the gut microbiota has been proven to be promising to prevent and treat different diseases, pharmaceutical and food industries would be interested in the potential therapeutic approaches described before. For instance, Seres health and Rebiotix companies are working on developing a defined microbial cocktail and a standardized commercially prepared FMT, respectively. These therapeutic approaches are intended to treat CDI, and that can be used as an alternative for FMT. Synthetic microbial communities designed for transplants are expected to meet production, mode of action and safety standards (Orenstein et al., 2015; van der Lelie et al., 2017). For instance, Seres health developed SER-109, a novel biological agent proposed to restore the balance in the gut microbiome, promoting resistance to pathogenic invaders like *C. difficile* (Khanna et al., 2016). Seres health also developed SER-287 for the treatment of IBD and in specific ulcerative colitis (Inflammatory Bowel Disease |Seres Therapeutics, 2017). Rebiotix commercially developed RBX2660, a mix of live human microbes for effective treatment of recurrent CDI (Ramesh et al., 2016). Moreover, other formulations including strains belonging to Clostridia classes IV and XIVa were designed to modulate the immune response (Atarashi et al., 2013). The original community of 17 strains (VE202) was developed by Vendanta Biosciences and Johnson and Johnson, and has provided an effective treatment for autoimmune disorders (Reardon, 2014; Ratner, 2015; van der Lelie et al., 2017).

### Technical Challenges

Several challenges concerning the stability of the probiotic during the probiotic production are still unsolved. Microorganisms require strict conditions to grow, such as specific nutritional media and environmental conditions (suitable temperature, pH, water activity, oxygen content, among others). The product manufacturing and storage processes may impact the viability of the bacterial strains, influencing probiotic stability and properties. In addition, it is fundamental to consider the viability of the probiotics after consumption. Bacterial strains should remain viable at sufficient numbers through the gastrointestinal tract (GIT) passage. Therefore, the selection of optimal culture medium and cell protectants is crucial to enhance the efficacy of the probiotic product. Moreover, as most probiotic strains are strict anaerobes or facultative anaerobes, oxygen permeation into carriers should be reduced, or oxygen scavengers should be introduced to reduce the redox potential (Shah et al., 2010). Probiotic bacteria can also be protected by microencapsulation, which has been proposed to improve the stability of the strains and can adapt to the GIT conditions (Heidebach et al., 2012). Nowadays, yogurts and fermented milk are the best-established vehicles for probiotics in the market. However, some probiotic strains are sensitive to the different conditions in fermented products, like oxygen and pH, which can, in turn, affect the stability of probiotics through post-acidification during their storage in the fridge. To minimize this phenomenon, strains that lack the ability to post-acidify should be selected (Damin et al., 2008). As a result, this can cause an economic burden for manufacturers, limiting the addition of probiotics in different products (Gueimonde and Sánchez, 2012). Furthermore, manufacturing the probiotic product in a reproducible manner is a critical aspect (Paulo Sousa e Silva and Freitas, 2014). Several attempts to fix the number of viable probiotic strains throughout the products (Shah et al., 2010) have been attempted, to no avail.

### Regulatory Challenges

Probiotics are classified in different categories across countries. Their names and use as functional foods may vary according to different systems. For instance, probiotics fall in the Qualified Presumption of Safety (QPS) list provided by the European Food Safety Authority (EFSA) and referred to as functional foods since there was no legal definition for probiotics. The market for probiotics as functional foods expanded, as a result of probiotic food products like yogurts and fermented milk (Baldi and Arora, 2015), containing conventional LAB. The QPS list is periodically updated according to the safety assessment of the biological products recommended to be added, and not all can be approved (Scientific Opinion on The Maintenance of The List of QPS Biological Agents Intentionally Added to EFSA Panel on Biological Hazards (BIOHAZ), 2013; Ricci et al., 2017). A similar system applies in the United States as Generally Recognized as Safe (GRAS) products should be approved by the FDA. However, if a probiotic is used as a dietary supplement in the United States, then it is considered as “food” and should be regulated by the Dietary Supplement Health and Education Act (DSHEA). If the probiotic was considered to have therapeutic purpose, the probiotic drug should be proven to be safe and effective to be approved by the FDA. Nevertheless, for both the FDA and EFSA, probiotics cannot be used in health claims. On the other hand, Japan acts as a global market leader, where probiotics are considered as both foods and drugs. According to the Japanese regulations, probiotic products are in different category than foods and Foods for Specific Health Uses (FOSHU). Efficacy claims for probiotic products are prohibited on the labeling until the product gets the permission from the Ministry of Health and Welfare (MHLW) to be considered FOSHU, for which efficacy and safety validation is mandatory. FOSHU categorizes the food claims according to the scientific evidence and the strength of the supporting data provided. The government then divided the FOSHU health claims into subcategories, in which their effect could be in GIT, metabolism, cholesterol moderation, or bone health. Japanese regulations also approve new health claims on a regular basis (Baldi and Arora, 2015).

As the definition and classification of probiotics by regulatory agents throughout the world is different, the status of probiotic products is still uncertain. Thus, reservations about probiotic products claims may arise among regulatory bodies, producers, and consumers. Since the probiotic concept is invading the world, further investigation for probiotic traits is needed. Moreover, most probiotics only include LAB, which possess limited phylogenetic diversity and functionality. Hence, critical update of the screenings required by regulatory agents is urgently needed.

### Medical Application

Despite the different studies and outcomes of FMT, FDA approval in North America has not been granted. At the beginning, FMT was considered as investigational new drugs (INDs), and FDA authorization was mandatory. Currently, patients unresponsive to standard antibiotic CDI therapies can opt for FMT after completing an informed consent, where they are notified that FMT is still under investigation. However, SERES 109 and RBX2660 have been granted the Orphan Drug designation by the FDA (Rebiotix Media, 2015; Seres Therapeutics, 2015). As for the EMA in Europe, the use of FMT for the treatment of CDI has not been yet regulated (van Nood et al., 2014; Lowes, 2016). Yet, FMT is regularly applied to curb infections across Europe, and it is considered in clinical trials for many other pathologies. In the search for safe FMT alternatives, research on microbiotic medicinal products (MMP) is in full development and novel applications are continuously being considered. These MMP developments require novel views and strategies from the scientific world, the industry, the medical field, and the regulatory bodies. In this context, platforms like the Pharmabiotic Research Institute have been created, to facilitate discussion between different stakeholders (Pharmabiotic Research Institute, 2017). Overall, additional research needs to be conducted before using FMT alternatives containing characterized microbial communities and next generation probiotics, to guarantee their safety and reproducible efficacy.

## Conclusion

FMT may be replaced with a characterized multispecies bacterial mixture that can be safer, free of allergens or viruses, and capable of treating CDI. With the current *in vitro* and *in vivo* data, next generation probiotics hold promise to treat diverse medical conditions, and they can be more effective than single or multi strains of the commercial probiotics. Moreover, several different strains with proven health benefits can also be considered candidates for next generation probiotics and other microbiota-based drugs. However, additional research is required for an increased understanding of the interactions among those strains, aiming at producing a successful therapeutic formulation. Research should be conducted to demonstrate whether these probiotics can be applicable to humans, as safety assessments have only been completed in animals. Effective carriage of bacterial strains in food matrices is critical for survival. Thus, optimisation of the growing conditions, and even encapsulation must be considered to promote delivery and release of the live product in the colon. The development of next generation probiotics and MMPs hold promise for innovation in both the food/feed sector and the pharmaceutical industry. A close interaction between academia, industry and regulatory agencies is essential for developing safe and health-promoting products, as both prophylactic and therapeutic strategies.

## Author Contributions

Conceived and designed the review: REH, EH-S, and TVW. Funding acquisition: TVW. Wrote the paper: REH. Reviewed the manuscript: REH, EH-S, and TVW.

## Conflict of Interest Statement

The authors declare that the research was conducted in the absence of any commercial or financial relationships that could be construed as a potential conflict of interest.
